# Two-Point Magnitude MRI for Rapid Mapping of Brown Adipose Tissue and Its Application to the R6/2 Mouse Model of Huntington Disease

**DOI:** 10.1371/journal.pone.0105556

**Published:** 2014-08-21

**Authors:** Katrin S. Lindenberg, Patrick Weydt, Hans-Peter Müller, Axel Bornstedt, Albert C. Ludolph, G. Bernhard Landwehrmeyer, Wolfgang Rottbauer, Jan Kassubek, Volker Rasche

**Affiliations:** 1 Department of Neurology, Ulm University, Ulm, Germany; 2 Department of Internal Medicine II, Ulm University, Ulm, Germany; 3 Core Facility Small Animal Imaging, Ulm University, Ulm, Germany; Faculty of Biology, Spain

## Abstract

The recent discovery of active brown fat in human adults has led to renewed interest in the role of this key metabolic tissue. This is particularly true for neurodegenerative conditions like Huntington disease (HD), an adult-onset heritable disorder with a prominent energy deficit phenotype. Current methods for imaging brown adipose tissue (BAT) are in limited use because they are equipment-wise demanding and often prohibitively expensive. This prompted us to explore how a standard MRI set-up can be modified to visualize BAT *in situ* by taking advantage of its characteristic fat/water content ratio to differentiate it from surrounding white fat. We present a modified MRI protocol for use on an 11.7 T small animal MRI scanner to visualize and quantify BAT in wild-type and disease model laboratory mice. In this application study using the R6/2 transgenic mouse model of HD we demonstrate a significantly reduced BAT volume in HD mice vs. matched controls (n = 5 per group). This finding provides a plausible structural explanation for the previously described temperature phenotype of HD mice and underscores the significance of peripheral tissue pathology for the HD phenotype. On a more general level, the results demonstrate the feasibility of MR-based BAT imaging in rodents and open the path towards transferring this imaging approach to human patients. Future studies are needed to determine if this method can be used to track disease progression in HD and other disease entities associated with BAT abnormalities, including metabolic conditions such as obesity, cachexia, and diabetes.

## Introduction

Brown adipose tissue (BAT) is a key tissue for the regulation of whole-body energy metabolism [1]. Through the expression of mitochondrial uncoupling protein 1 (UCP-1), which allows proton leakage across the inner mitochondrial membrane, BAT is capable of dissipating energy in the form of heat. BAT is thus the principal effector tissue of adaptive, non-shivering thermogenesis. A series of breakthrough positron emission tomography (PET) and computed tomography (CT) imaging studies has recently shown BAT deposits even in adult humans [2]–[5]. This resulted in a renewed interest in the role of BAT especially for metabolic conditions such as obesity and cachexia, thereby creating the need for appropriate imaging technologies [6]. Large-scale studies aiming at the structural and functional characterization of BAT by PET/CT in human adults are still scarce [4], [6], [7]. Factors contributing to this limitation include the high costs for the required tracers, safety concerns about the radiation involved, and the required activation of BAT either via cold-exposure or pharmacologically.

Huntington disease (HD), an autosomal dominant adult-onset neurodegenerative disease caused by the abnormal expansion of the CAG-repeat tract in the huntingtin gene, represents an area of research where these new developments are of particular interest [8]. In addition to the classic triad of neuro-psychiatric symptoms, i.e. movement disorder, cognitive decline and psychiatric abnormalities, HD patients display a progressive energy deficit that is of high clinical and scientific relevance [9]. This energy deficit is recapitulated in transgenic mouse models of HD and this led to the implication of brown fat dysfunction in HD pathogenesis, even before BAT-activity in human adults was discovered [10], [11]. Selected transgenic mouse models of HD therefore provide a useful model system to study disease-related BAT dysfunction *in vivo*.

Hamilton and colleagues performed an in-depth analysis of the magnetic resonance (MR) signatures of BAT and white adipose tissue (WAT) [12]. They reported several key MR properties that are different between BAT and WAT, including fat fraction, changes in the T1 relaxation rate of the water-bond protons, and the degree of lipid saturation. The use of the histological and physiological properties of BAT has the major advantage of not requiring prior stimulation and has been applied for fat characterization by means of magnetic resonance spectroscopy (MRS) and chemical-shift imaging (CSI) [13]–[15].

With the exception of intermolecular zero-quantum coherences spectroscopy aiming for assessment of the intravoxel distribution of fat and water spins [16] and blood oxygen level dependent MRI [17], the majority of published fat fraction imaging methods by MRI relies on direct fat/water quantification as initially introduced by Dixon and colleagues in 1984 [18]. Based on the original publication, several modifications of the technique have been proposed [19]. Although in principle off-resonance contributions can be compensated by the multi-point techniques, additional non-static phase errors and the need for phase unwrapping quite frequently introduce errors in the resulting fat/water maps and may cause exclusion of data [15] especially at ultrahigh field strength.

The goal of this work was to establish and validate a simplified two-point technique for direct visualization of BAT, using the difference between the magnitude of images obtained with in-phase and opposed-phase condition. The applicability of the proposed technique was tested pre-clinically in an HD disease model (R6/2 transgenic mouse). HD appears of special interest since brown fat abnormalities are of emerging interest in the investigation of the HD energy deficit, and the clear genetic pathogenesis of HD provides an excellent basis for future mechanistic studies in transgenic model systems and facilitates validation in human patients and translational approaches.

## Results

**Figure 1:**
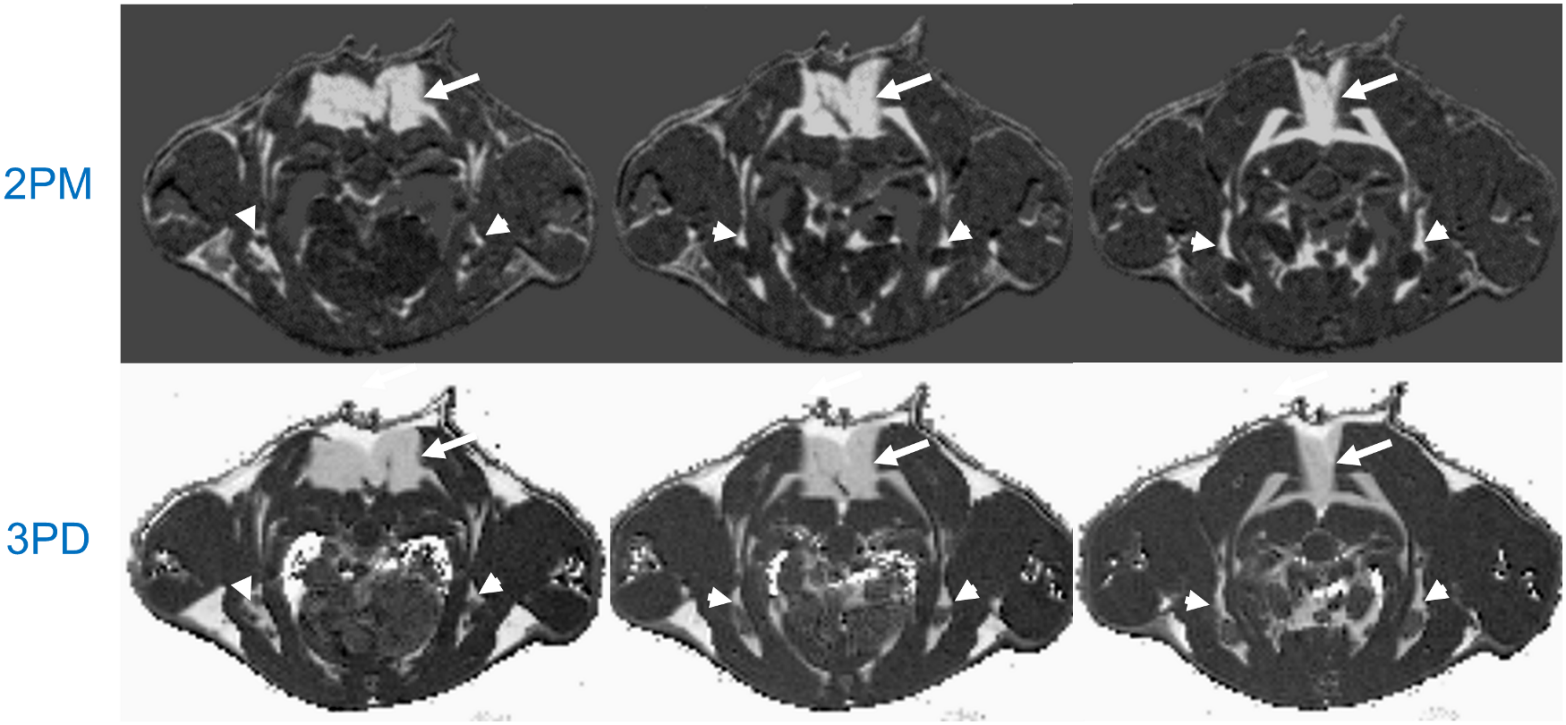
Direct comparison of the novel two-point magnitude method (2PM) and the conventional three-point phase-based Dixon method (3PD) in a wild-type mouse. Interscapular BAT deposits (arrows) as well as smaller pads of BAT (arrowheads) can be appreciated. Please note the excellent agreement between the two methods. Due to the suppression of pure fat and water voxels in the 2PM technique, a clear improvement of contrast between WAT and BAT areas can be appreciated with the 2PM technique.

All scans could be completed successfully in the investigated cohorts. Automatic thresholding applying the Otsu method was effective in full suppression of background signal as well as pixels with low signal intensities as e.g. present in the lung and cortical bone. **Figure 1** shows a direct *in vivo* comparison of the 2PM approach with the conventional 3PD method. The image intensity in the 2PM approach is linearly related to the fat-water fraction with a maximum at equal magnetization of fat- and water spins. Since BAT is expected to show an almost equal magnetization of fat- and water spins, BAT presents as bright signal intensity in the final 2PM images. Water- and fat-only voxels show a clearly reduced intensity. The signal in the fat fraction images derived from the 3PD method scales linearly with the fat fraction in the magnetization. Water-only voxels appear dark, fat-only voxels appear bright, and BAT voxels present with intermediate signal intensity. The direct comparison shows an excellent resemblance of the resulting BAT distribution between the two approaches. The interscapular fat deposits (Figure 1, arrows) can be clearly appreciated as well as some smaller pads of BAT extending anteriorly and bilaterally around a large vessel (Figure 1, arrowheads). Note that both methods show a similar sensitivity to partial volume effects.

**Figure 2:**
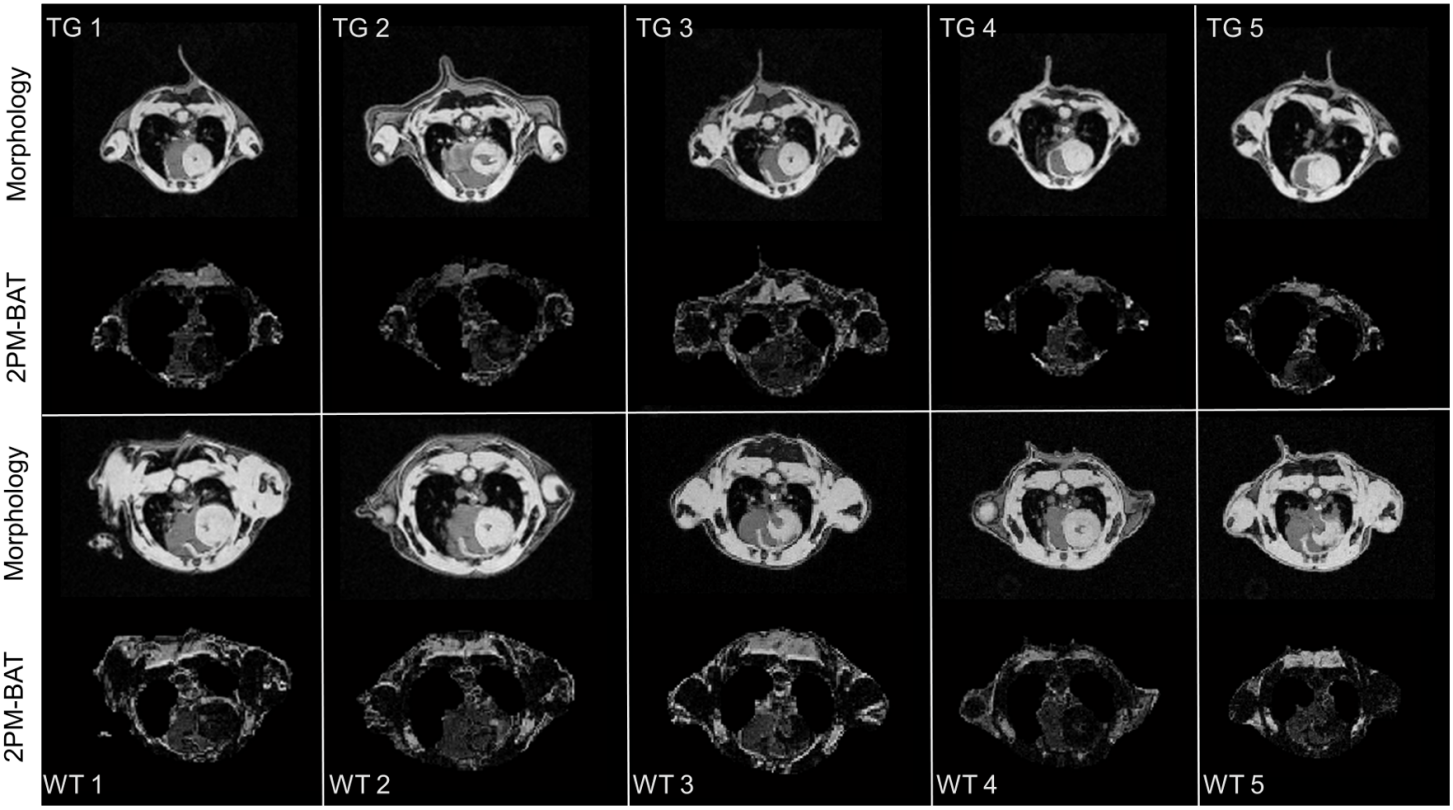
Comparison of morphological images and resulting BAT-weighed images from R6/2-transgenic (TG) and corresponding wild-type (WT) mice. Note the overall reduced tissue volume in the R6/2 mice. The slices were selected to comprise the shoulder region where the prominent interscapular BAT pad is located.

An overview of the BAT pattern for the wild-type and transgenic R6/2 mice is provided in **Figure 2** for a slice centered at the location of the prominent interscapular BAT. As expected and as described previously [20], [21], the reduced body size and general organ atrophy of R6/2 mice vs. wild-type controls is immediately evident.

**Figure 3:**
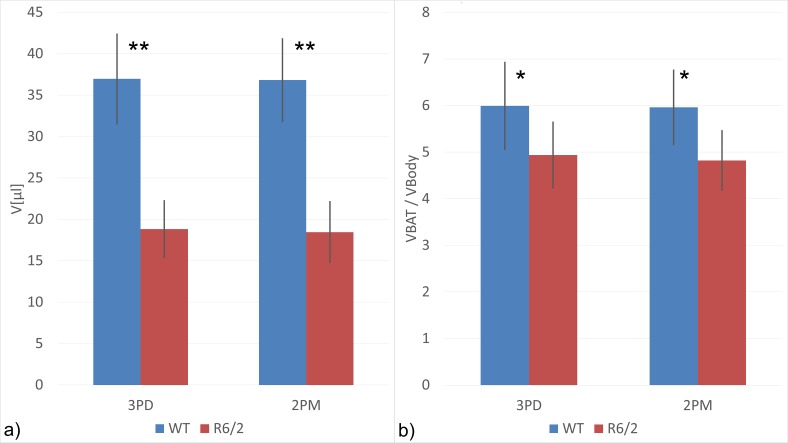
Total BAT volumes a) and BAT volumes normalized to total body volume b) for female HD-transgenic (R6/2) and matched wild-type control mice. (n = 5 per group; *P<0.05, **P<0.01; unrelated Wilcoxon Rank sum test; columns (error bars) represent means (standard deviation)).

Even though the spatial resolution was lowered for reduction of the acquisition time, the BAT distribution can be readily retrieved from the resulting 2PM images. Semi-automatic quantification of the BAT volumes showed a highly significant positive correlation of the 2PM and the conventional 3PD method (R = 0.976, p<0.01, Spearmans’s rho). Further analysis allowed for direct and quantitative comparison of BAT volumes in the region of interest between HD mice and matched wild-type controls. As shown in **Figure 3a**, R6/2 mice have a greatly reduced BAT volume (P<0.01) of 18.46 µl (min: 14.18 µl, max: 23.85 µl) ml vs. 36.82 µl (min: 32.34 µl, max 45.40 µl) in controls. The difference is blunted but still manifest and statistically significant (P<0.05) when normalization to total body volume as a correction for the different body sizes is introduced (**Figure 3b**).

## Discussion

The study evaluates a non-invasive method, which allows for *in vivo* and *ex vivo* BAT volume quantification from magnitude images. The intrinsic insensitivity of the technique to any kind of phase errors introduced by system imperfections should cause an at least similar reproducibility of 2PM as the more established fat-fraction methods [14], [22], [23]. Since the approach is based on a conventional FLASH technique, it can be readily used on any conventional MRI scanner without further modification of acquisition methods. In comparison to other MRI-based BAT-imaging techniques, the advantage of the 2PM method is the straightforward approach, which does not require any special expertise in advanced MRI techniques or image processing. The technique can be readily modified further to provide blood oxygen level dependent contrast. Khanna et al. [17] showed that T2* weighting times of about 2 ms are sufficient for the assessment of BAT activity. For magnetic field strengths between 1.5 and 11.7 T as used for clinical and pre-clinical imaging, one of the required in- and opposed-phase echo times can be chosen close to the suggested value. Furthermore, the 2PM method can also easily be adapted to the suggested asymmetric spin echo acquisition if the required echo times cannot be met due to gradient power constraints. In this context the possibility of intrinsic identification of the BAT location from the scans obtained at normal body temperature is advantageous. Compared to the more complex intermolecular zero-quantum MRI [16], [24], 2PM is hampered by partial volume effects similar to the fat fraction methods. However, slightly higher spatial resolution can be realized without significant scan time penalties since images with only two different echo times are required for providing the BAT information.

By use of our MRI-based tissue analysis, we show that R6/2 mice have significantly reduced BAT, even when corrected for reduced overall body size. This finding provides a plausible structural explanation for the striking cold-sensitive phenotype of R6/2 mice and other HD models [10], [11]. The results are also in accordance with earlier reports of structural BAT abnormalities in transgenic mouse models of HD, including the R6/2 strain, all of which were obtained through non-survival conventional tissue dissection [10], [11], [25], [26]. Importantly, our MRI-method now provides a practical strategy for monitoring BAT structure *in vivo* and longitudinally in the same individual animal.

While we focused our present study on the R6/2 mice, other transgenic mouse strains with a cold-sensitive body temperature phenotype such as the N171-82Q model of HD [10] and the mutant SOD1 model of amyotrophic lateral sclerosis [27] should also be investigated with respect to BAT structure, distribution and function *in vivo*. We would like to emphasize that this approach can naturally be applied to other diseases, e.g. obesity and diabetes. The results further suggest that this non-invasive methodology can be readily applied to human patients and control subjects. However, the different distribution of the BAT in humans especially in small pads as e.g. as in perivascular tissue may in general limit the direct BAT imaging approaches independent of the imaging technique used. In this context the improved scan efficiency of the proposed 2PM method may be beneficial due to better spatial resolution without sacrificing scan time compared to multi-point Dixon methods.

An important limitation of the data set presented here is the focus on a single fat pad in the interscapular region. We selected this region as it is recognized as the largest and most prominent BAT depot in rodents [28]. This choice permitted reliable anatomical localisation of the region of interest. It also allowed for direct comparison with the histological data from HD models, as to the best of our knowledge all the BAT tissue dissection studies in HD used interscapular BAT. This focus, however, did preclude characterisation of the BAT distribution patterns. As we know from imaging studies, fat distribution can vary in specific disease states [29] and how such distribution changes play out in BAT pathology remains to be investigated. As a further limitation, our study was not designed to detect the so called beige or *brite* (*br*own-in-wh*ite*) adipocytes [30] but in principle the presented tissue characterisation algorithm can be utilized to do so, if sufficient spatial resolution was achieved. Further in vivo studies with cold exposure have to be performed since the assessment of activated BAT has not been tested in this study. Also, since the method presented here relies on the characteristic relative fat/water content of BAT the use under conditions where the BAT composition is altered, e.g. starvation, require adjustments that were beyond the scope of this study.

In summary, we present a straightforward technique for assessing BAT structure with conventional MRI set-ups. Further research and development is necessary to integrate functional information, e.g. in the form of blood oxygen level dependent signal, into the MRI data acquisition protocol. The non-invasive *in vivo* character of the methodology allows longitudinal studies that were not previously possible. In a first application of the new methodology, we were able to show that symptomatic R6/2 mice have a reduced BAT volume. This finding provides insight into to the energy deficit in HD and highlights the importance of the peripheral tissue pathology for the understanding of the HD phenotype [31].

## Materials and Methods

### Theory

Depending on the environment, differently bond ^1^H protons experience different resonance frequencies caused by the shielding effects of the electrons. This characteristic chemical shift can be utilized for tissue characterization and structural analysis by MRS and is frequently applied to chemical analysis [32]–[34] in biomedical research. For ^1^H proton spectroscopy, the reference with 0 ppm chemical shift is defined by tetramethysilane (TMS) and the chemical shift of different organic compounds is defined relative to TMS. Chemical shifts of fat bond ^1^H protons range from 0.9 ppm (−(CH_2_)_n_−CH_3_) to 5.3 ppm (−CH = CH−) and water (H_2_O) showing a chemical shift of 4.7 ppm.

Hamilton et al. [12] investigated the MR properties of WAT and BAT. Important findings included alteration of endogenous biochemical characteristics as water – fat fraction (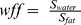, with 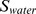 being the MR signal from water-bond protons and 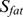 the respective MR signal from fat-bond protons), T1 relaxation times, and lipid saturation. From the investigated parameters 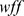 showed the most pronounced difference between WAT (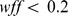) and BAT (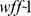). The higher amount of water in BAT has driven the application of fat fraction quantification to BAT quantification in mice [14] as well as in humans [15], [22], [35].

Fat fraction quantification demands accurate knowledge of the fat and water content in each voxel. Separation of water and fat can be achieved by Dixon techniques [18], [23], [36]. Dixon methods rely on the phase differences between MRI images obtained with different echo times (TE). The phase differences are introduced by the chemical shift of the different compounds. The MRI signal 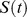
from voxels containing different compounds results as
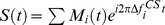
(1)with 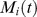 being the magnetization of the *i*-th component at time *t* and 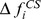 it’s chemical shift. Although, different proton moieties are present, the fat contribution in BAT and WAT is dominated by the methylene protons (1.3 ppm), followed by methyl (0.9 ppm) and allylic protons (2.0 ppm). Considering water and methylene protons, only, Eq. 1 simplifies to
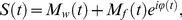
(2)with 
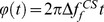
 being the phase between water and fat-bond protons. Theoretically, the fat (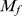) and water (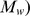 image can be easily reconstructed from the sum (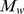) and the difference (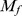) of gradient echo images acquired in in-phase (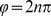) and opposed-phase (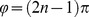) condition as suggested as the original two-point Dixon method [18]. A major limitation for the two-point Dixon method arises from local off-resonances, introducing an additional TE-dependent phase term and Eq. 2 must be rewritten as:
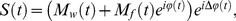
(3)with 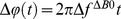 being the off-resonance introduced phase error at time t. To compensate for the additional phase term, off-resonance maps and phase unwrapping techniques have to be used. Several techniques have been introduced so far for compensation of the off-resonance induced phase errors [36]–[38]. All techniques require additional measurements, which prolong the overall acquisition time. Off-resonance contributions can be compensated by the multi-point techniques, additional non-static phase errors and the need for phase unwrapping frequently introduce errors in the resulting fat/water maps especially at ultrahigh field strength.

**Figure 4:**
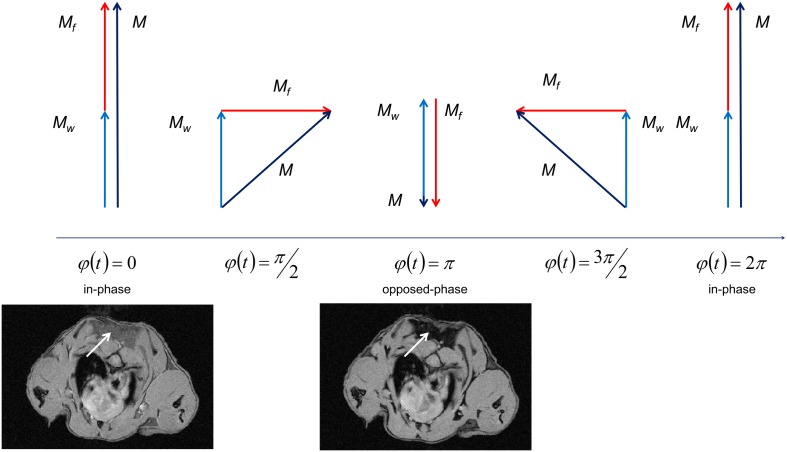
Resulting magnetization M (black) in a voxel containing almost equal concentration of water- (*M_w_*, blue) and fat- (*M_f_* , red) bond protons for different phase differences. Respective MR images of the mouse neck in axial orientation are exemplarily shown for in-phase (left) and opposed-phase (right) condition. The reduction of signal intensity in the interscapular BAT (arrows) is clearly appreciable.

In this study, a magnitude-based approach is introduced suggesting the use of the difference of the magnitude of two images acquired with echo times (TE) values for BAT volume quantification. The approach is based on the assumption that a major difference between WAT, normal biological tissue, and BAT results from the almost equal concentration of water- and fat-bond protons in BAT. The magnitude of 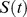 results from [Eq. 3] as
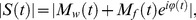
(4)where the phase 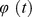 between the water- and fat-bond protons cause an intensity modulation depending on the magnetization of the fat 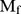 and water 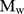 with almost complete signal cancellation at opposed phase condition in case of equal water and fat contributions as shown in **Figure 4**. For background suppression of voxels containing a single tissue, only, the final BAT signal is calculated according to:
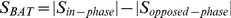
(5)


### Animal groups

The suggested two-point magnitude (2PM) technique was initially evaluated in vivo in direct comparison with the three-point Dixon (3PD) technique in a group of three wild-type mice (C57/B6, 12 months, female). All in vivo data acquisition was carried out under isoflurane anesthesia (3% for induction and 1.5% for maintenance).

Applicability to cohort studies was evaluated in the R6/2 transgenic mouse model compared to sex-matched wild-type litter mates. The R6/2 mouse is among the most commonly used transgenic models of HD [20], [39]. They display cold-intolerance highly suggestive of BAT dysfunction, and structural abnormalities of white and brown adipose tissue are well-documented [10], [25], [40]. Female mice were used at 12 weeks, an age when this model is clearly symptomatic. Assuming a volume change in the order of 50% in the R6/2 group, to achieve a statistical power of 80% sample sizes of 5 animals per group were required. Power was calculated with nQuery (version 10). To exclude any impact of respiratory and cardiac motion artifacts in this feasibility study, the animals were sacrificed (CO_2_ inhalation) immediately prior to the MRI investigation.

In vivo animal experiments were performed in accordance with German animal protection laws and had been approved by the national animal board (TVA 1001, “Etablierung Kleintierbildgebung an 3T und 11.7T”, Regierungspräsidium Baden-Württemberg, Tübingen, Germany).

### MRI protocol

The proposed two-point magnitude method (2PM) was implemented and evaluated against a 3PD method based on data acquired on a dedicated high-field 11.7T small animal MRI system (BioSpec 117/16, Bruker, Germany, Ettlingen). All data were acquired applying a 4cm quadrature volume resonator.

For initial in vivo evaluation (C57/B6) of the 2PM methods, a high-spatial resolution spoiled fast low-angle shot gradient echo technique (HR-FLASH, Δr = 100×100×500 µm^3^) was applied. Data were acquired at five different echo times (TE = 1.203 ms, 1.313 ms, 1.459 ms, 1.507 ms, 1.751 ms), yielding phase shifts of -π/6, π/2, 7 π/6, π, 2 π respectively. The applicability to cohort studies (R6/2, wild-type) was tested with a reduced spatial resolution protocol (LR-FLASH, Δr = 120×200×500 µm^3^) to ensure rapid quantification of the BAT volumes. In all animals, the complete neck area was covered by acquiring 9 slices of 500 µm slice thickness with 1 mm spacing. Acquisition time T_ACQ_ for the acquisition of a single TE resulted to T_ACQ_ = 3m: 50s (LR-FLASH) and T_ACQ_ = 6m 24s (HR-FLASH). Excitation angle α and repetition time TR were as α = 15° and TR = 75 ms.

### Data processing

**Figure 5:**
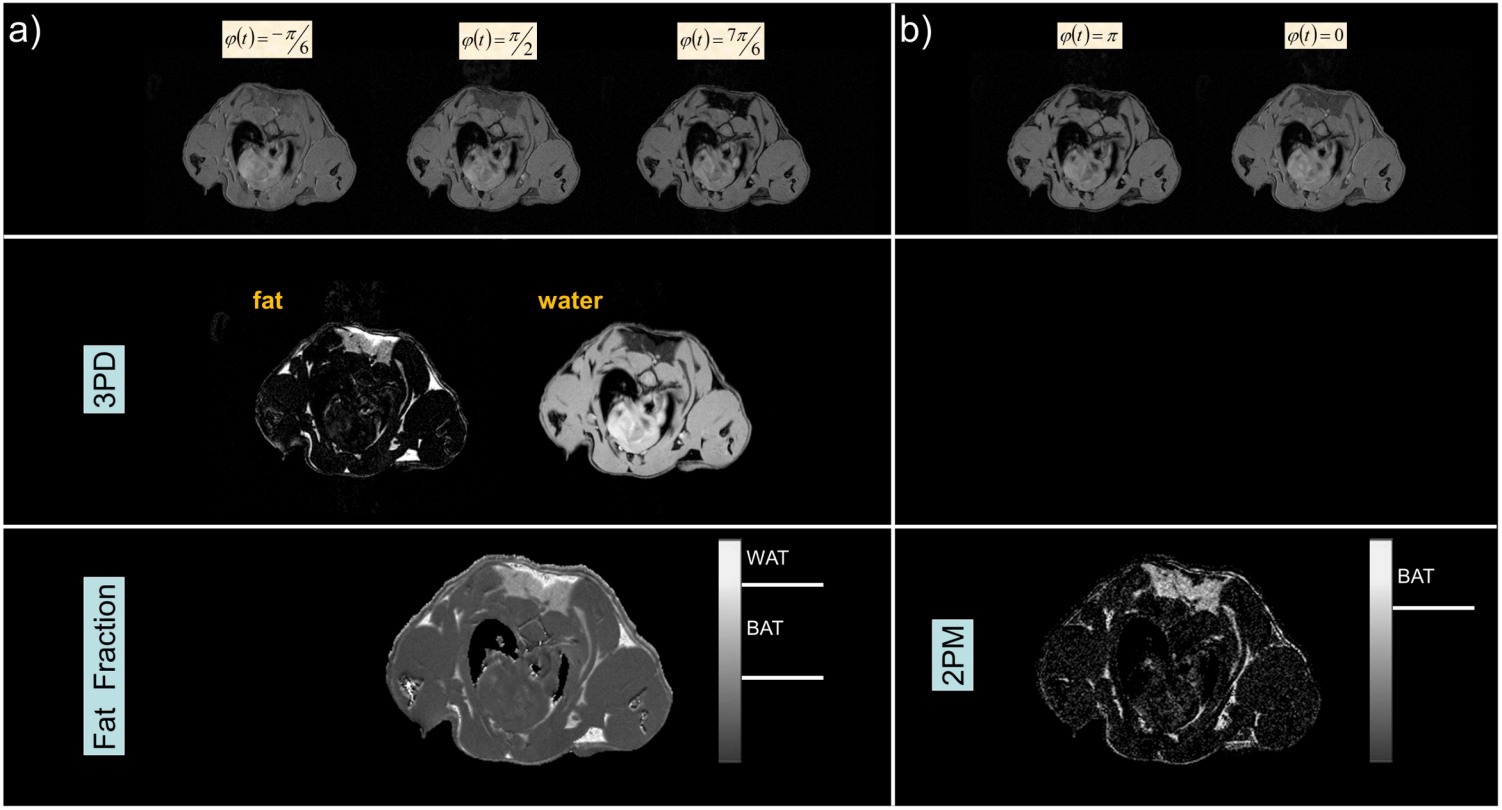
Comparison of the fat-fraction method based on a three-point Dixon approach (3PD, a), and the proposed two-point magnitude based method (2PM, b). The fat-fraction a) and BAT b) image are normalized from 0 to 1. The grey bars indicate the respective grey values used as threshold for WAT (>0.8, 3PD) and for BAT (>0.4 and <0.8, 3PD; >0.7, 2PM). WAT cannot be identified by the 2PM method, since signal from voxels containing only fat or water will be cancelled out during the involved subtraction.

After acquisition, the data was transferred to an independent workstation and further processed for BAT quantification. Fat fraction images were derived from the images acquired with -π/6, π/2, 7π/6 phase shifts applying a multi-point Dixon method. For ensuring high quality of the fat fraction maps, the “fat-water toolbox” (http://ismrm.org/workshops/FatWater12/data.htm) provided by the ISMRM was applied for all calculations. For evaluation of the suggested two-point magnitude technique, the difference of the magnitude of the images obtained with in-phase and opposed-phase condition was calculated. An overview of the two approaches is provided in **Figure 5**. For noise reduction prior to further analysis, a 3×3 point medium filter was applied. Background signal was removed by thresholding the input data. The respective threshold value was derived from the in-phase images according to the Otsu threshold algorithm [41].

Volume quantification of the BAT was performed using a semi-automatic segmentation tool (http://www.itksnap.org/, [42]). Regions of BAT were identified in a slice-by-slice fashion according to the mean normalized grey value 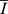 with 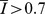 for the 2PM method and 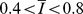 for the 3PD method. Falsely identified regions due to partial volume effects were removed manually for both approaches. The evaluation was performed by an experienced MRI scientist (VR, >15 yrs in research). Quantification was performed blinded for the two investigated approaches.

After semi-automated classification, resulting BAT volumes were compared between 2PM and 3PD as well as between the HD-transgenic mice and the wild-type controls. Correlation of the volumes derived from the investigated methods was assessed applying a Spearman’s rho test and the significance of BAT volume changes between the different groups was assessed by an unrelated Wilcoxon rank sum test. P-values below 0.05 were considered significant. All statistical analysis was performed with SPSS 21.0 (IBM Corp, Armonk, NY).

## References

[1] Lee P, Swarbrick MM, Ho KK (2013) Brown adipose tissue in adult humans: a metabolic renaissance. Endocr Rev 34: 413–438. 23550082 10.1210/er.2012-1081

[2] Nedergaard J, Bengtsson T, Cannon B (2007) Unexpected evidence for active brown adipose tissue in adult humans. Am J Physiol Endocrinol Metab 293: E444–452. 17473055 10.1152/ajpendo.00691.2006

[3] Cypess AM, Lehman S, Williams G, Tal I, Rodman D, et al (2009) Identification and importance of brown adipose tissue in adult humans. N Engl J Med 360: 1509–1517. 19357406 10.1056/NEJMoa0810780 PMC2859951

[4] van Marken Lichtenbelt WD, Vanhommerig JW, Smulders NM, Drossaerts JM, Kemerink GJ, et al (2009) Cold-activated brown adipose tissue in healthy men. N Engl J Med 360: 1500–1508. 19357405 10.1056/NEJMoa0808718

[5] Virtanen KA, Lidell ME, Orava J, Heglind M, Westergren R, et al (2009) Functional brown adipose tissue in healthy adults. N Engl J Med 360: 1518–1525. 19357407 10.1056/NEJMoa0808949

[6] Bauwens M, Wierts R, van Royen B, Bucerius J, Backes W, et al (2014) Molecular imaging of brown adipose tissue in health and disease. Eur J Nucl Med Mol Imaging 41: 776–791. 24509875 10.1007/s00259-013-2611-8

[7] Wijers SL, Saris WH, van Marken Lichtenbelt WD (2010) Cold-induced adaptive thermogenesis in lean and obese. Obesity (Silver Spring) 18: 1092–1099. 20360754 10.1038/oby.2010.74

[8] Rona-Voros K, Weydt P (2010) The role of PGC-1alpha in the pathogenesis of neurodegenerative disorders. Curr Drug Targets 11: 1262–1269. 20840068 10.2174/1389450111007011262

[9] Mochel F, Haller RG (2011) Energy deficit in Huntington disease: why it matters. J Clin Invest 121: 493–499. 21285522 10.1172/JCI45691 PMC3026743

[10] Weydt P, Pineda VV, Torrence AE, Libby RT, Satterfield TF, et al (2006) Thermoregulatory and metabolic defects in Huntington’s disease transgenic mice implicate PGC-1alpha in Huntington’s disease neurodegeneration. Cell Metab 4: 349–362. 17055784 10.1016/j.cmet.2006.10.004

[11] Chaturvedi RK, Calingasan NY, Yang L, Hennessey T, Johri A, et al (2010) Impairment of PGC-1alpha expression, neuropathology and hepatic steatosis in a transgenic mouse model of Huntington’s disease following chronic energy deprivation. Hum Mol Genet 19: 3190–3205. 20529956 10.1093/hmg/ddq229 PMC2915615

[12] Hamilton G, Smith DL Jr, Bydder M, Nayak KS, Hu HH (2011) MR properties of brown and white adipose tissues. J Magn Reson Imaging 34: 468–473. 21780237 10.1002/jmri.22623 PMC3146031

[13] Lunati E, Marzola P, Nicolato E, Fedrigo M, Villa M, et al (1999) In vivo quantitative lipidic map of brown adipose tissue by chemical shift imaging at 4.7 Tesla. J Lipid Res 40: 1395–1400. 10428975

[14] Hu HH, Smith DL Jr, Nayak KS, Goran MI, Nagy TR (2010) Identification of brown adipose tissue in mice with fat-water IDEAL-MRI. J Magn Reson Imaging 31: 1195–1202. 20432356 10.1002/jmri.22162 PMC2924147

[15] Rasmussen JM, Entringer S, Nguyen A, van Erp TG, Guijarro A, et al (2013) Brown adipose tissue quantification in human neonates using water-fat separated MRI. PLoS One 8: e77907. 24205024 10.1371/journal.pone.0077907 PMC3813555

[16] Branca RT, Warren WS (2011) In vivo brown adipose tissue detection and characterization using water-lipid intermolecular zero-quantum coherences. Magn Reson Med 65: 313–319. 20939093 10.1002/mrm.22622 PMC3021650

[17] Khanna A, Branca RT (2012) Detecting brown adipose tissue activity with BOLD MRI in mice. Magn Reson Med 68: 1285–1290. 22231619 10.1002/mrm.24118 PMC3326213

[18] Dixon WT (1984) Simple proton spectroscopic imaging. Radiology 153: 189–194. 6089263 10.1148/radiology.153.1.6089263

[19] Hu HH, Bornert P, Hernando D, Kellman P, Ma J, et al (2012) ISMRM workshop on fat-water separation: insights, applications and progress in MRI. Magn Reson Med 68: 378–388. 22693111 10.1002/mrm.24369 PMC3575097

[20] Mangiarini L, Sathasivam K, Seller M, Cozens B, Harper A, et al (1996) Exon 1 of the HD gene with an expanded CAG repeat is sufficient to cause a progressive neurological phenotype in transgenic mice. Cell 87: 493–506. 8898202 10.1016/s0092-8674(00)81369-0

[21] Sathasivam K, Hobbs C, Turmaine M, Mangiarini L, Mahal A, et al (1999) Formation of polyglutamine inclusions in non-CNS tissue. Hum Mol Genet 8: 813–822. 10196370 10.1093/hmg/8.5.813

[22] Chen YI, Cypess AM, Sass CA, Brownell AL, Jokivarsi KT, et al (2012) Anatomical and functional assessment of brown adipose tissue by magnetic resonance imaging. Obesity (Silver Spring) 20: 1519–1526. 22343821 10.1038/oby.2012.22 PMC4383098

[23] Hu HH, Kan HE (2013) Quantitative proton MR techniques for measuring fat. NMR Biomed 26: 1609–1629. 24123229 10.1002/nbm.3025 PMC4001818

[24] Branca RT, Zhang L, Warren WS, Auerbach E, Khanna A, et al (2013) In vivo noninvasive detection of Brown Adipose Tissue through intermolecular zero-quantum MRI. PLoS One 8: e74206. 24040203 10.1371/journal.pone.0074206 PMC3769256

[25] Johri A, Calingasan NY, Hennessey TM, Sharma A, Yang L, et al (2012) Pharmacologic activation of mitochondrial biogenesis exerts widespread beneficial effects in a transgenic mouse model of Huntington’s disease. Hum Mol Genet 21: 1124–1137. 22095692 10.1093/hmg/ddr541 PMC3277311

[26] Ho DJ, Calingasan NY, Wille E, Dumont M, Beal MF (2010) Resveratrol protects against peripheral deficits in a mouse model of Huntington’s disease. Exp Neurol 225: 74–84. 20561979 10.1016/j.expneurol.2010.05.006

[27] Dupuis L, Oudart H, Rene F, Gonzalez de Aguilar JL, Loeffler JP (2004) Evidence for defective energy homeostasis in amyotrophic lateral sclerosis: benefit of a high-energy diet in a transgenic mouse model. Proc Natl Acad Sci U S A 101: 11159–11164. 15263088 10.1073/pnas.0402026101 PMC503756

[28] Cannon B, Nedergaard J (2004) Brown adipose tissue: function and physiological significance. Physiol Rev 84: 277–359. 14715917 10.1152/physrev.00015.2003

[29] Lindauer E, Dupuis L, Muller HP, Neumann H, Ludolph AC, et al (2013) Adipose Tissue Distribution Predicts Survival in Amyotrophic Lateral Sclerosis. PLoS One 8: e67783. 23826340 10.1371/journal.pone.0067783 PMC3694869

[30] Rosenwald M, Wolfrum C (2014) The origin and definition of brite versus white and classical brown adipocytes. Adipocyte 3: 4–9. 24575363 10.4161/adip.26232 PMC3917931

[31] van der Burg JM, Bjorkqvist M, Brundin P (2009) Beyond the brain: widespread pathology in Huntington’s disease. Lancet Neurol 8: 765–774. 19608102 10.1016/S1474-4422(09)70178-4

[32] Jahnke W, Widmer H (2004) Protein NMR in biomedical research. Cellular and Molecular Life Sciences 61: 580–599. 15004697 10.1007/s00018-003-3382-3 PMC11138659

[33] Pellecchia M, Sem DS, Wuthrich K (2002) NMR in drug discovery. Nat Rev Drug Discov 1: 211–219. 12120505 10.1038/nrd748

[34] Marion D (2013) An introduction to biological NMR spectroscopy. Mol Cell Proteomics 12: 3006–3025. 23831612 10.1074/mcp.O113.030239 PMC3820920

[35] Hu HH, Yin L, Aggabao PC, Perkins TG, Chia JM, et al (2013) Comparison of brown and white adipose tissues in infants and children with chemical-shift-encoded water-fat MRI. J Magn Reson Imaging 38: 885–896. 23440739 10.1002/jmri.24053 PMC3664653

[36] Ma J (2008) Dixon techniques for water and fat imaging. J Magn Reson Imaging 28: 543–558. 18777528 10.1002/jmri.21492

[37] Glover GH (1991) Multipoint Dixon technique for water and fat proton and susceptibility imaging. J Magn Reson Imaging 1: 521–530. 1790376 10.1002/jmri.1880010504

[38] Bley TA, Wieben O, Francois CJ, Brittain JH, Reeder SB (2010) Fat and water magnetic resonance imaging. J Magn Reson Imaging 31: 4–18. 20027567 10.1002/jmri.21895

[39] Pouladi MA, Morton AJ, Hayden MR (2013) Choosing an animal model for the study of Huntington’s disease. Nat Rev Neurosci 14: 708–721. 24052178 10.1038/nrn3570

[40] Fain JN, Del Mar NA, Meade CA, Reiner A, Goldowitz D (2001) Abnormalities in the functioning of adipocytes from R6/2 mice that are transgenic for the Huntington’s disease mutation. Hum Mol Genet 10: 145–152. 11152662 10.1093/hmg/10.2.145

[41] Otsu N (1979) Threshold Selection Method from Gray-Level Histograms. Ieee Transactions on Systems Man and Cybernetics 9: 62–66.

[42] Yushkevich PA, Piven J, Hazlett HC, Smith RG, Ho S, et al (2006) User-guided 3D active contour segmentation of anatomical structures: significantly improved efficiency and reliability. Neuroimage 31: 1116–1128. 16545965 10.1016/j.neuroimage.2006.01.015

